# Bioorthogonal Cyclization Reactions for the Synthesis of Cytotoxic Drugs in Cancer Treatment

**DOI:** 10.1021/acsomega.5c07644

**Published:** 2025-11-20

**Authors:** Gean M. Dal Forno, Brenno A. D. Neto, Josiel B. Domingos

**Affiliations:** † Laboratory of Biomimetic Catalysis (LaCBio), Department of Chemistry, 28117 Federal University of Santa Catarina (UFSC), Campus Trindade, 88040-900 Florianópolis, SC, Brazil; ‡ Laboratory of Medicinal and Technological Chemistry, 28127 University of Brasília, Chemistry Institute (IQ-UnB), Campus Universitário Darcy Ribeiro, 70910-900 Brasília, Distrito Federal, Brazil; § Molecular Sciences Graduate Program, Universidade Estadual de Goiás, 75132-400 Anápolis, GO, Brazil

## Abstract

Cyclization reactions play a central role in the synthesis of heterocycles, many of which display potent cytotoxicity and are actively being developed as anticancer agents. Recent breakthroughs have enabled these transformations to occur in situ within living systems, thereby advancing novel bioorthogonal strategies for spatially controlled cancer therapies. This strategy enhances therapeutic efficacy by promoting the localized formation of active drugs at tumor sites, significantly reducing systemic toxicity and off-target effects. Herein, we review recent advances in the use of bioorthogonal cyclization reactions to generate cytotoxic (hetero)­cycles, including triazoles, benzotriazoles, phenanthridines, phenanthridinium salts, quinoxalines, coumarins, naphthalenes, and pyrrolizidine derivatives, directly within cancer cells. We examine the cyclization reaction classes employed, the triggers (e.g., transition metals, light, and reactive oxygen species), underlying mechanisms, and bioorthogonal strategies enabling these transformations. This Mini Review offers a focused overview of state-of-the-art methods and their therapeutic applications and highlights the transformative potential of in situ cyclization as a platform for developing next-generation, highly efficient anticancer therapies.

## Graphical abstract

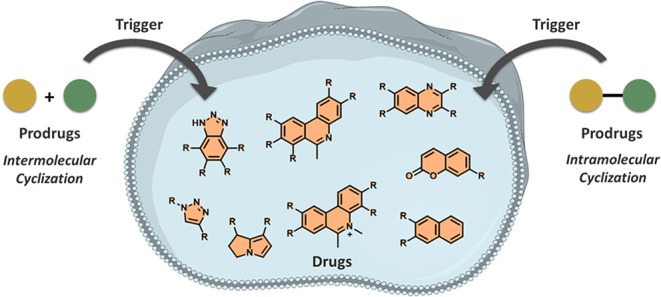

## 1 Introduction

The translation of cyclization reactions for the synthesis of heterocycles, traditionally conducted under controlled laboratory conditions (e.g., concentration, temperature, and optimized solvent systems), into living systems via bioorthogonal chemistry marks a paradigm shift in the design of therapeutic agents.^1^ These well-established reaction parameters face major challenges in the highly aqueous, heterogeneous, and dynamic environment of living organisms.^2^ Nonetheless, the advent of robust bioorthogonal strategies now enables such transformations to proceed effectively in living cells, paving the way for site-specific drug generation and precision therapies. This potential was notably exemplified by the 2022 Nobel Prize in Chemistry, awarded to Carolyn Bertozzi, alongside K. Barry Sharpless and Morten Meldal, for pioneering bioorthogonal reactions and copper-catalyzed azide–alkyne cycloadditions (CuAAC), which laid the foundation for the in vivo synthesis of cytotoxic triazoles.^3^ The in situ synthesis of cytotoxic drugs for cancer treatment encompasses two primary approaches: intermolecular cyclization (Figure 1A), which necessitates the interaction of two distinct precursors to produce a cytotoxic heterocycle, and intramolecular cyclization, wherein a single precursor undergoes rearrangement to form the active compound (Figure 1B). Although prior reviews have addressed bioorthogonal approaches involving bond cleavage or bond formation,^4–10^ few have systematically focused on the in situ generation of heterocycles specifically for cancer therapy. This review aims to bridge the gap between synthetic methodology and clinical relevance by outlining the key reaction classes, mechanistic insights, and biomedical applications of in situ heterocycle formation. It also highlights the emerging design principles for next-generation systems (Figure 1C), thereby guiding future development of targeted chemotherapeutic agents.

**Figure 1:**
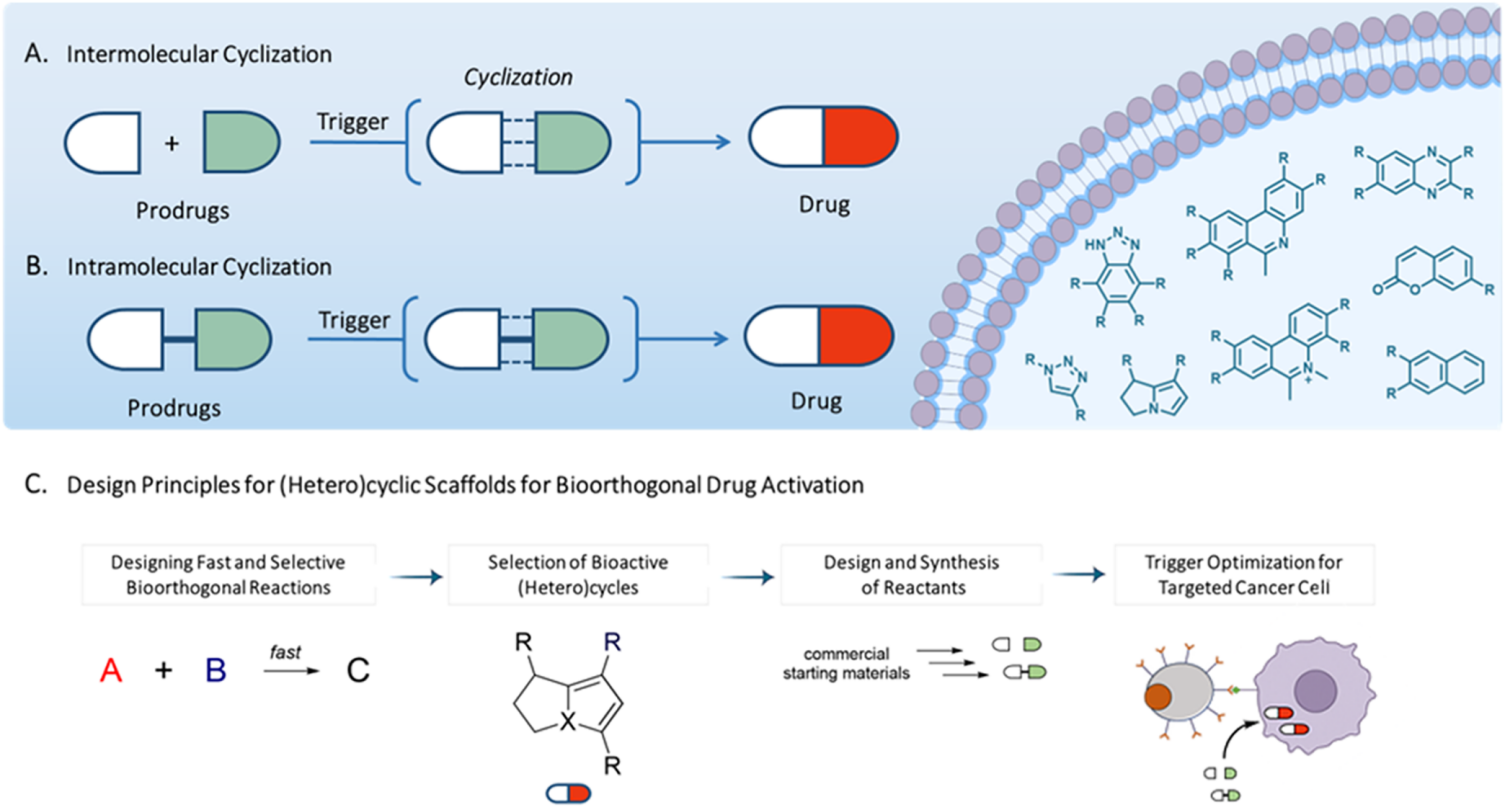
In situ synthesis of cytotoxic agents for cancer therapy and representative examples of (hetero)­cycles generated through intracellular cyclization. (A) Intermolecular cyclization. (B) Intramolecular cyclization. (C) Key design principles for bioorthogonal cyclization reactions leading to cytotoxic (hetero)­cycles. Figure adapted from Servier Medical Art (https://smart.servier.com), licensed under CC BY 4.0.

## 2 Classes of Bioorthogonal Cyclization Reactions

Several structurally distinct heterocycles have been synthesized in situ within biological systems through bioorthogonal cyclization reactions. These include diverse frameworks such as 1,2,3-triazoles, benzotriazoles, phenanthridines, phenanthridinium salts, quinoxalines, coumarins, naphthalenes, and pyrrolizidine derivatives. Despite their structural variability, all of these (hetero)­cycles share a common trait: they can be generated from relatively innocuous precursors and undergo selective activation under biological conditions. The sections that follow detail representative examples of each class, highlighting the catalytic systems, activation mechanisms, and antitumoral efficacy of the resulting structures.

### 2.1 1,2,3-Triazoles

The first click reaction, copper­(I)-catalyzed azide–alkyne cycloaddition (CuAAC), has garnered broad interest due to its capacity to construct complex molecules in high yields, with rapid kinetics, and minimal byproducts, using straightforward and nontoxic building blocks. However, the use of Cu­(I) in living systems is limited by its inherent toxicity. To overcome this, biocompatible ligands and nanostructures have been developed to both mitigate cytotoxicity and enhance catalytic efficiency, enabling CuAAC to proceed in cellular and animal models. Notably, triazoles synthesized intracellularly by CuAAC, often inspired by natural products such as Combretastatin A4 and Resveratrol, have demonstrated micromolar cytotoxicity and promising anticancer activity.

#### 2.1.1 Combretastatin A4 Analog

In 2016, Bradley and co-workers pioneered the in situ synthesis of a triazole-based anticancer agent (compound 3) via CuAAC from two nontoxic precursors, azide (1) and alkyne (2) (Figure 2).^11^ The reaction was catalyzed by heterogeneous, biocompatible Cu nanoparticles embedded in TentaGel resin (160.4 μm). The resulting triazole (3) showed strong cytotoxicity against SKOV-3 and HeLa cells, it is a micromolar cytotoxic analog of Combretastatin A4 (CA4) (4), a tubulin polymerization inhibitor which exhibits cytotoxicity against several cancer cell lines.^12,13^ Notably, the reaction occurred extracellularly due to the size of the catalyst, and mass analysis confirmed the presence of both triazole and its precursors inside cells postreaction.

**Figure 2:**
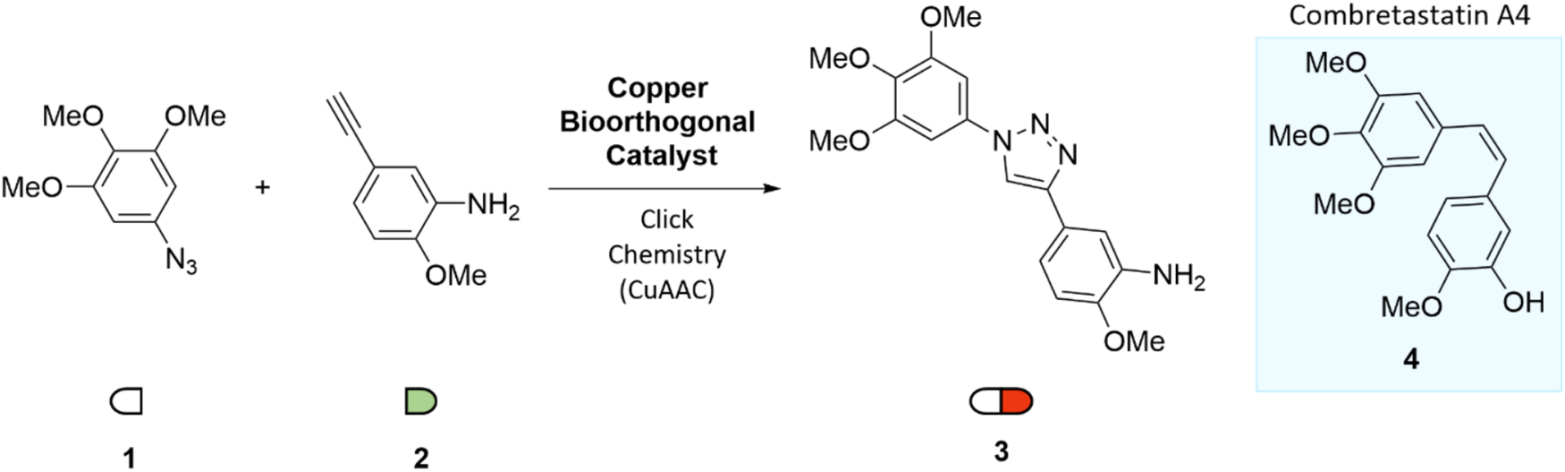
Synthesis of anticancer 1,2,3-triazole (3) analog of Combretastatin A4 (4) by CuAAC.

Following this initial report, several research groups developed diverse strategies to synthesize the triazole-based anticancer agent (3) directly within cancer cells or animal models. In 2018, Zhang and co-workers reported its intracellular formation using nanocopper-doped cross-linked lipoic acid nanoparticles, which enabled CuAAC to proceed inside cells with only 15 ppm of copper.^14^ They subsequently introduced a copper­(I)-chelated micellar nanocatalyst bearing surface-localized copper. In HeLa cells, this system yielded a 2-fold increase in cytotoxicity compared to the direct administration of compound (3) at 50 μM, attributed to enhanced cellular uptake of the precursors and elevated intracellular triazole formation, with just 20 ppm of copper.^15^ In 2021, the same group reported a dual-nanocapsule platform in which both precursors (1 and 2) and the copper catalyst were separately encapsulated in cross-linked lipoic acid nanocapsules, denoted as (1 + 2)@cLANCs and Cu@cLANCs, respectively.^16^ These nanostructures enabled staggered administration and selective release at tumor sites, triggered by elevated glutathione (GSH) levels of the tumor microenvironment. Upon activation, the cLANCs released their payloads to facilitate the in situ formation of triazole (3) via CuAAC.

In 2023, Chen and co-workers developed an ultrasound-triggered copper nanocatalyst for the spatially and temporally controlled synthesis of triazole (3), combined with ROS generation for synergistic antitumor effects in vitro and in murine xenograft models.^17^ The system consisted of ultrasmall poly­(acrylic acid)-coated copper nanocomplexes activated by ultrasound-induced Cu­(II)/Cu­(I) redox cycling, which simultaneously promoted catalytic turnover and ROS production.

In 2025, Zhao group reported a cancer cell-specific strategy for in situ triazole (3) generation using a copper–bipyridine single-site catalyst embedded in a cerium-based metal–organic framework (MOF).^18^ This nanocatalyst exhibited a high CuAAC activity, as confirmed by single-molecule fluorescence microscopy. Selective targeting to cancer cells was achieved via two features: (i) surface camouflage with vesicles derived from 4T1 or A549 cancer cell membranes and (ii) Cu­(II)-to-Cu­(I) activation mediated by elevated intracellular GSH. In addition to the catalysis, the MOF displayed oxidase and peroxidase-like activities, contributing to ROS production and further enhancing the anticancer response.

#### 2.1.2 Resveratrol Analog

In 2006, Genazzani and co-workers reported a series of triazole-containing analogs of resveratrol (8), in which the central double bond was replaced with a 1,2,3-triazole via a CuAAC reaction.^19^ A decade later, in 2016, Qu and co-workers selected compound (7) for in situ synthesis in mitochondria using a functionalized heterogeneous copper catalyst embedded in a metal–organic framework (MOF) (Figure 3). The MOF stabilized the copper nanoparticles and was functionalized with triphenylphosphonium (TPP), a well-established mitochondria-targeting group, to ensure mitochondrial localization of the catalyst.^20^ Azide (5) and alkyne (6) precursors exhibited negligible cytotoxicity up to 25 μM. In contrast, intracellular synthesis of triazole (7) using the MOF–Cu–TPP catalyst significantly reduced cell viability to below 30% and induced both mitochondrial damage and apoptosis. Notably, direct administration of compound (7) resulted in minimal cytotoxicity and no oxidative stress. This strategy was also evaluated in a murine tumor model, where the MOF–Cu–TPP system proved biocompatible and effectively reduced tumor growth via in situ generation of compound (7).

**Figure 3:**
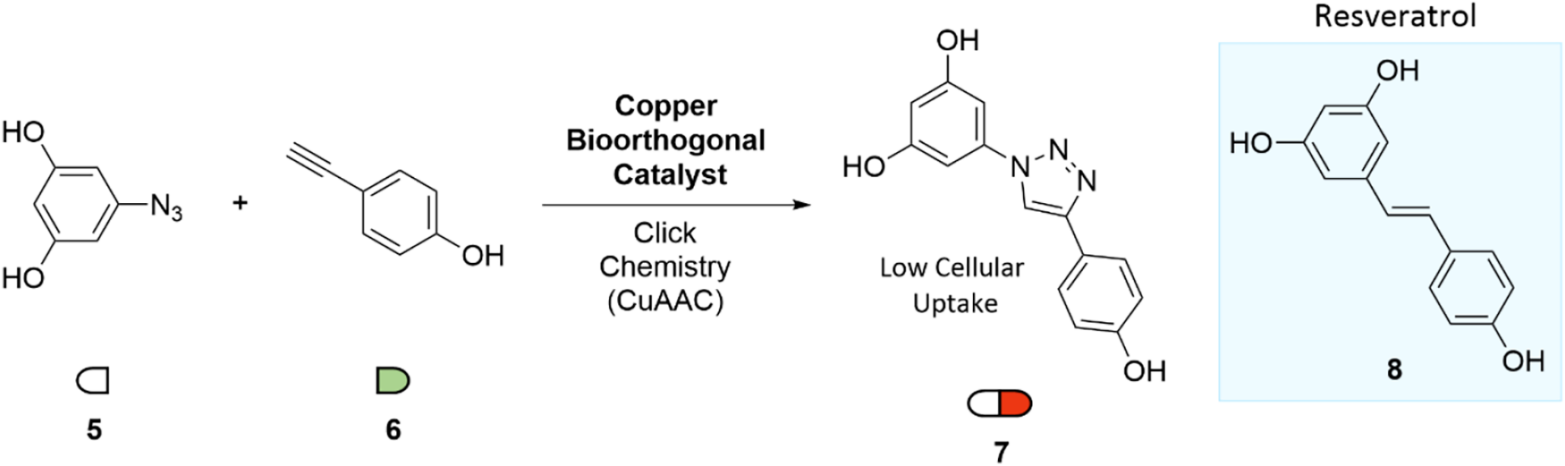
Intracellular synthesis of low-membrane-permeable anticancer 1,2,3-triazole (7) analog of Resveratrol by CuAAC.

In subsequent years, Qu’s group continued to explore innovative strategies for the intracellular synthesis of triazole (7). In 2020, they developed a biocompatible heterogeneous copper catalyst activated by near-infrared (NIR) light, enabling photoresponsive CuAAC reactions in vivo. Upon NIR irradiation, the system displayed both photothermal and photodynamic effects.^21^ In vivo application in tumor-bearing mice confirmed the biosafety and antitumoral efficacy of in situ triazole (7) synthesis mediated by the MCNs–Cu + NIR system. In 2022, they introduced a DNA-functionalized copper platform capable of targeting bioorthogonal catalysis in living systems. This strategy enhanced therapeutic efficacy while reducing off-target effects in animal models.^22^ In 2023, a copper-free strategy was reported in which the reactants (5) and (6), along with sodium ascorbate, were coencapsulated in adenosine triphosphate (ATP) aptamer-functionalized MOF nanoparticles (MOF–NPs).^23^ Upon interaction with ATP and Zn^2+^, the nanoparticles disassembled, releasing the payloads. The sodium ascorbate reduced endogenous Cu­(II) to catalytically active Cu­(I), triggering intracellular CuAAC and the formation of compound (7). In 2024, the group reported a ZIF-8-based MOF system designed to mimic copper chaperones, selectively binding Cu^2+^ via imidazole-copper interactions. Once internalized by cancer cells, the nanoparticles released copper ions in response to the acidic tumor microenvironment, enabling in situ catalysis.^24^ After endocytosis by cancer cells, the nanoparticles release copper ions in response to the acidic tumor microenvironment. That same year, they also developed a membrane-coated nanoparticle for the targeted delivery of disulfiram (DSF), a copper ionophore. This system induced synergistic cell death via combined cuproptosis and CuAAC-mediated synthesis of compound (7).^25^

In 2023, Bai and co-workers reported the membrane-localized synthesis of low-permeability triazoles using a macromolecular copper catalyst embedded in cationic dense-shell nanoparticles (DSNPs).^26^ These polymeric phosphonium-functionalized nanoparticles protect the catalyst from deactivation and exhibit a high affinity for cell membranes. The system enabled CuAAC of hydrophobic anionic substrates on the membrane surface, facilitating enhanced cellular uptake of otherwise membrane-impermeable drugs. The authors proposed that this approach could be broadly applied for the on-membrane synthesis of hydrophilic drugs, improving therapeutic efficacy relative to direct drug administration.

#### 2.1.3 Cell Autophagy Induced by Triazole Formation

Autophagy-tethering molecules represent a novel class of compounds designed to modulate autophagy, a crucial catabolic process responsible for maintaining cellular homeostasis and adaptation to stress.^27^ Bai and co-workers extended the application of phosphonium-functionalized cationic dense-shell nanoparticles (PDSNPs) to mediate the intracellular delivery of large, anionic autophagic triazoles via CuAAC reactions.^26^ Using this strategy, they generated a phosphorylation-inducing chimeric molecule (11) directly on the cell membrane, thereby triggering autophagic pathways (Figure 4A). Evaluation of LC3 (a classical autophagy marker), mitochondrial membrane potential, and cell viability confirmed that in situ formation of the triazole elicited superior autophagic activity compared with direct application of the preformed compound, likely due to its limited membrane permeability.

**Figure 4:**
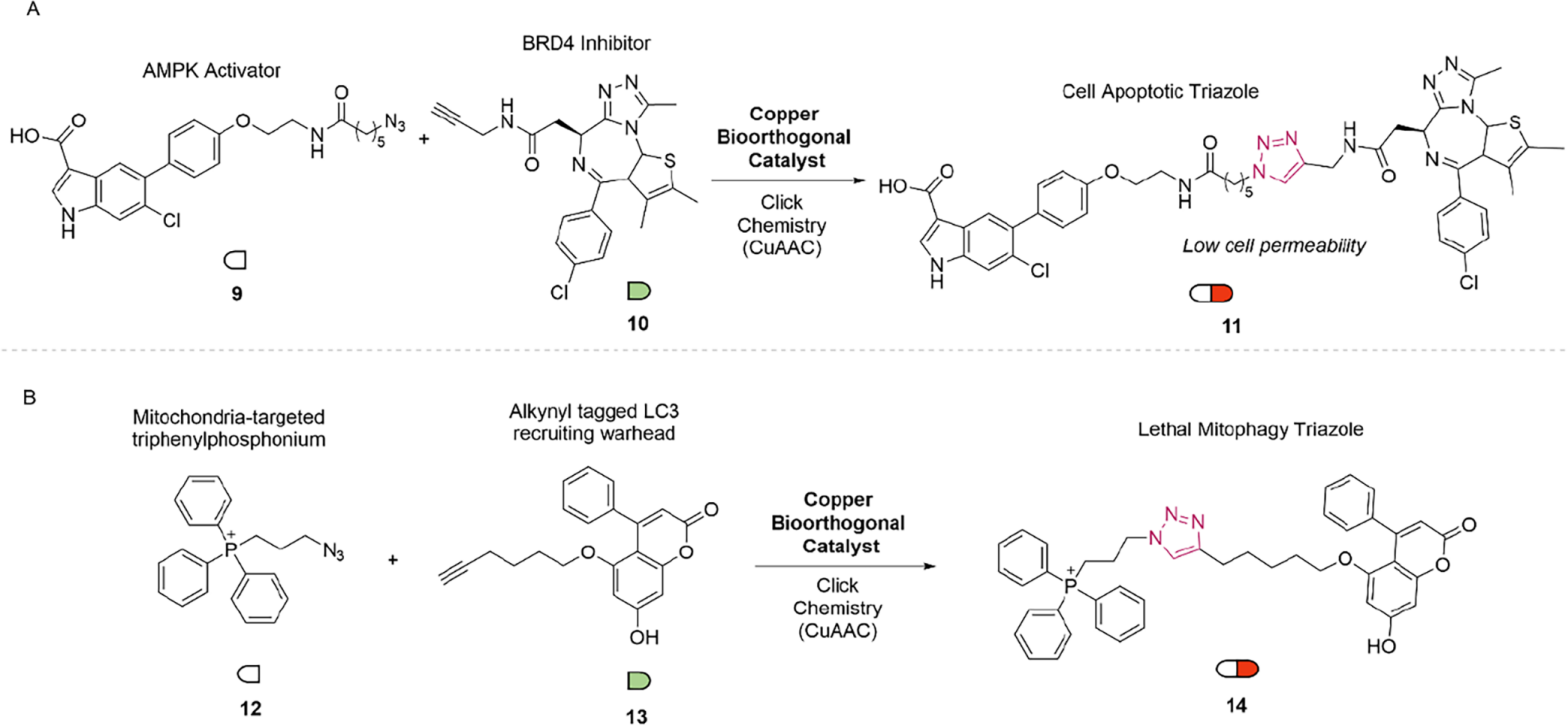
(A) CuAAC-mediated covalent ligation of an AMPK activator and BRD4 inhibitor to produce triazole kinase binder (**11**), an autophagy inducer. (B) CuAAC-mediated in situ generation of a lethal mitophagy agent (**14**).

Also in 2023, Xing and co-workers developed membrane-anchored copper catalysts with precise spatial control by employing a liposome fusion-based delivery platform, termed MAC-LiFT (Membrane-Anchored Catalysis via Liposome Fusion Transport).^28^ This method enabled the targeted deposition of catalytically active copper species on one or both leaflets of the cellular membrane. The study revealed that the inner membrane leaflet offers a more protective microenvironment for metal centers, which favors catalytic activity.

In parallel, Qu’s group repurposed their DNA-based copper catalyst platform to promote in situ synthesis of a lethal mitophagy inducer (14) (Figure 4B).^22^ This system employed an azide-tagged mitochondria-binding unit (12) and an alkyne-functionalized LC3-recruiting warhead (13), which underwent intracellular CuAAC to form compound (14).^29^ This bioorthogonal strategy promoted mitochondrial degradation (mitophagy), leading to autophagic cell death and significantly inhibiting tumor progression in B16-F10 melanoma-bearing mice.

Collectively, these studies underscore the therapeutic potential of in-cell triazole synthesis, whether at the membrane or intracellular level, to amplify cytotoxicity, particularly in cases where the isolated compounds possess low permeability or intrinsic activity. The examples summarized in Table 1 showcase a growing repertoire of copper-based bioorthogonal catalysts tailored for click chemistry in oncological settings. These strategies highlight advancements in catalyst design, intracellular localization, and selective activation for the CuAAC-mediated synthesis of cytotoxic 1,2,3-triazoles. By leveraging bioorthogonal catalysis to maximize intracellular Cu­(I) availability, these approaches demonstrate potent antitumoral effects, while minimizing off-target toxicity. Future efforts aimed at optimizing triazole scaffolds and catalyst delivery systems hold significant promise for advancing next-generation chemotherapeutics.^30^

**Table 1:** Copper Bioorthogonal Catalysts for Click Chemistry (CuAAC) in Cancer Treatment

triazole	strategy	catalyst activator^a^	key advantages	year	reference
3	extracellular biocompatible Cu-NPs embedded TentaGel resin		in situ cyclization	2016	^11^
3	nanocopper-doped cross-linked lipoic acid NPs		intracellular synthesis	2018	^14^
3	Cu(I)-chelated cross-linked micelle nanocatalyst		intracellular synthesis	2019	^15^
3	cross-linked lipoic acid nanocapsules with azide/alkyne reactants and Cu catalyst	GSH	intracellular delivery of 1/2 and synthesis of 3	2021	^16^
3	ultrasound-triggered Cu catalyst	US	synergic generation of ROS	2023	^17^
3	cancer cell-membrane decorated Cu-MOF catalyst	GSH	targeted intracellular synthesis	2025	^18^
7	mitochondria targeted Cu-MOF catalyst		mitochondrial synthesis	2016	^20^
7	mesoporous carbon nanospheres Cu catalyst	NIR	photodynamic and photothermal synergic effects	2020	^21^
7	DNA-based Cu nanocatalyst		targeted intracellular synthesis	2022	^22^
7	boosting endogenous Cu by MOFs	SA	intracellular drug synthesis by endogenous Cu(I)	2023	^23^
7	modulation of endogenous Cu trafficking by MOFs	GSH	intracellular drug synthesis by accumulation of endogenous Cu(I)	2024	^24^
7	cell-membrane-coated nanoparticle with azide/alkyne reactants and copper ionophore	GSH/H_2_O_2_	intracellular drug synthesis by boosting endogenous Cu(I) and synergic cuproptosis	2024	^25^
7 and 11	membrane-embedded copper catalyst		on-membrane synthesis of low-membrane-permeable triazoles	2023	^26^
11	membrane-anchored copper catalyst		synthesis with spatial control	2023	^28^
14	DNA-based Cu nanocatalyst		targeted mitochondrial synthesis	2023	^29^

a GSH: glutathione; US: ultrasound; NIR: near-Infrared light; SA: sodium ascorbate; H_2_O_2_: hydrogen peroxide.

### 2.2 Benzotriazoles

In 2024, Liu and co-workers reported a nitric oxide (NO)-triggered bioorthogonal cyclization reaction enabling the in situ synthesis of photothermal benzotriazoles within tumor tissues.^31^ The nanoplatform was constructed via nanoprecipitation by self-assembling the diamine precursor TTB-NH_2_ (15) with amphiphilic polyarginine (PArg). In this system, PArg serves a dual function: as a self-assembling nanocarrier for the o-phenylenediamine-based prodrug and as an endogenous NO donor. To enhance tumor specificity and systemic stability, the cationic complex TTB-NH_2_@PArg was further coated with hyaluronic acid (HA), yielding the tumor-targeting formulation TTB-NH_2_@HA-PArg (Figure 5A). Upon internalization by cancer cells, intracellular hydrogen peroxide (H_2_O_2_) levels trigger PArg decomposition, leading to localized NO release. This NO production initiates a rapid intramolecular cyclization of TTB-NH_2_ into the active benzotriazole TTB-AZO (16), a photothermal therapeutic agent (Figure 5B). Under 808 nm laser irradiation, TTB-NH_2_@PArg induced strong cytotoxicity in A549 and HeLa cells, reducing cell viability to 7%, while normal 3T3 cells remained largely unaffected, indicating selective antitumor activity. In vivo studies using the A549 tumor xenograft model demonstrated that TTB-NH_2_@HA-PArg combined with laser irradiation significantly inhibited tumor growth and prolonged animal survival.

**Figure 5:**
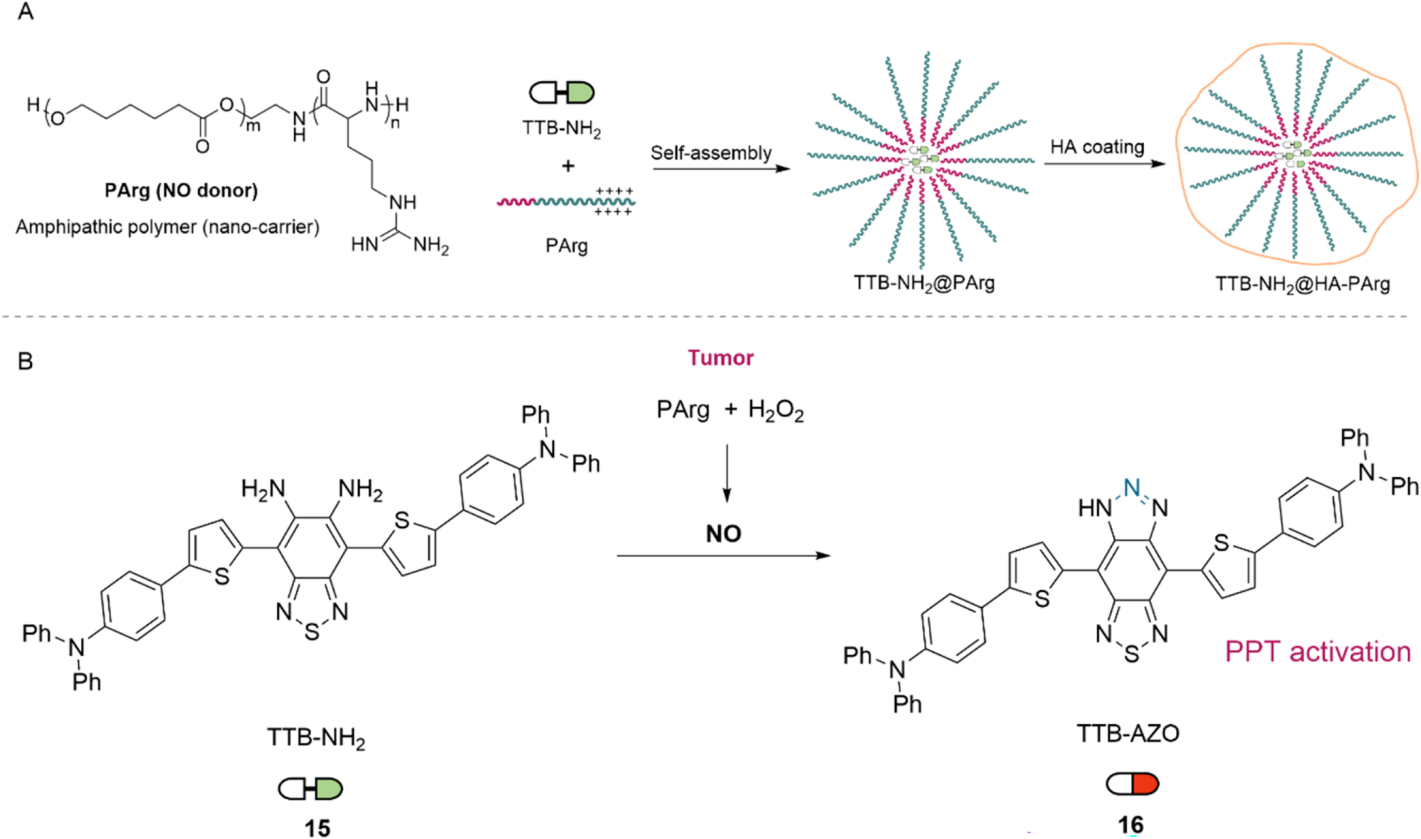
(A) Nanoassembly of cationic TTB-NH_2_@PArg and HA surface coating to afford tumor-targeted TTB-NH_2_@HA-PArg. (B) NO-mediated bioorthogonal bond-forming cyclization to generate photothermal benzotriazole TTB-AZO (**16**) in situ.

### 2.3 Phenanthridines

Phenanthridines are planar heterocyclic compounds frequently found in cytotoxic agents. Despite their potent biological activity, their clinical application remains limited due to systemic toxicity concerns.^32^ In 2021, Labruère and co-workers reported a strategy for the selective formation of phenanthridine derivatives in tumor cells using a bioorthogonal cyclization reaction triggered by elevated levels of reactive oxygen species (ROS) in the tumor microenvironment.^33^ The approach is based on nontoxic, ring-opened precursors bearing a vinylboronate functionality (ProToxPhen, 17), which undergo ROS-mediated activation. Upon oxidation, the vinylboronate group is converted to a ketone via enol-to-ketone tautomerization. This newly formed ketone then undergoes an intramolecular cyclization, yielding cytotoxic 6-methylphenanthridine (ToxPhen, 18) directly in situ (Figure 6). In KB epidermoid carcinoma cells, ToxPhen exhibited potent cytotoxicity with an IC_50_ of 0.67 μM, while its precursor ProToxPhen was essentially inactive (IC_50_ > 500 μM) in both 2D monolayer and 3D spheroid cultures. Under tumor-mimicking conditions, ProToxPhen was selectively activated in cancer cells to form fluorescent ToxPhen, demonstrating minimal activity in nontumoral tissues. This selectivity highlights the potential of ROS-triggered cyclization as a strategy to minimize off-target toxicity.

**Figure 6:**
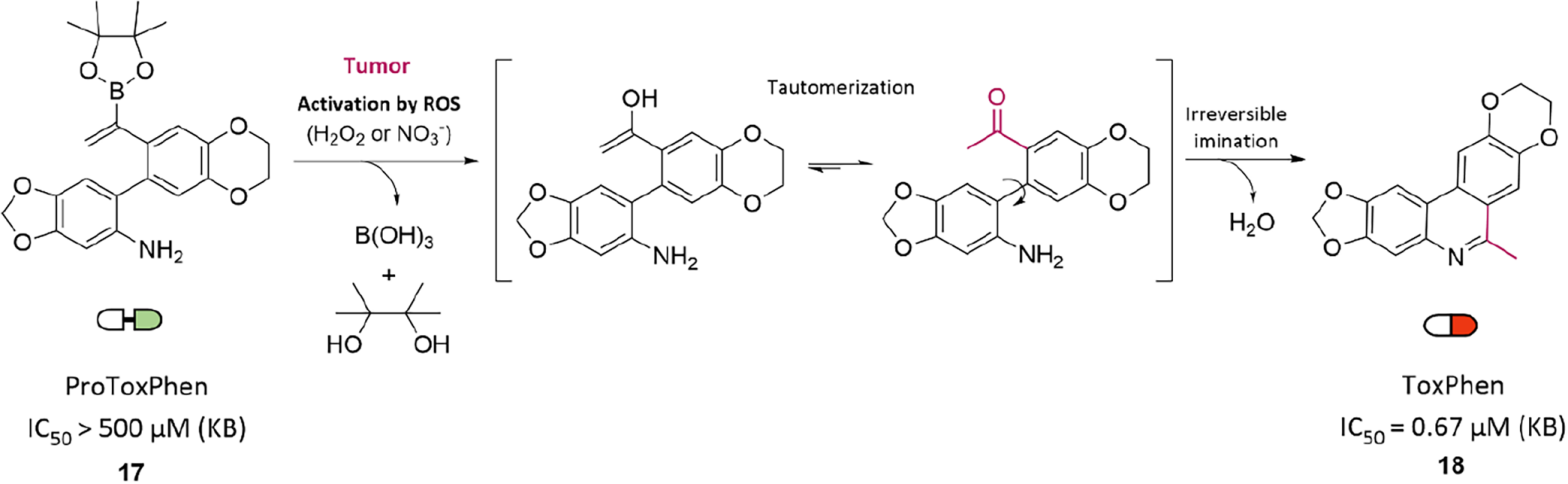
Synthesis of anticancer 6-methylphenanthridine (**18**, ToxPhen) via ROS-triggered bioorthogonal cyclization.

Looking forward, expanding the scope of stimuli-responsive triggers to activate phenanthridine-based prodrugs could further enhance the precision of these therapies. Recent efforts have described light- and metal-mediated methods for generating phenanthridines under biocompatible conditions; however, integration of these strategies with highly potent scaffolds such as ToxPhen remains an open avenue for further development.^34,35^

### 2.4 Phenanthridinium

Phenanthridinium derivatives represent a class of cationic heterocycles structurally related to naturally occurring alkaloids. These compounds have attracted significant attention due to their promising therapeutic properties, with several derivatives exhibiting strong cytotoxic activity against a variety of cancer cell lines.^32,36^ In 2021, Tanaka and co-workers reported a novel bioorthogonal cyclization reaction enabling the in situ synthesis of phenanthridinium derivatives. The transformation involves intramolecular C–N bond formation via activation of an alkene moiety, followed by a nucleophilic attack by a pendant amine, catalyzed by a gold complex (Figure 7).^37^ The researchers designed 2′-alkynyl-*N*-methyl-2-aminobiphenyl precursors, which, upon treatment with 0.25 mol % of a gold catalyst in PBS/dioxane (9:1) at 37 °C, yielded phenanthridinium derivatives with efficiencies reaching up to 99%. To enable application in biological environments, the gold catalyst was incorporated into albumin-based artificial metalloenzymes (ArMs), providing both catalytic stability against intracellular glutathione (GSH)-mediated deactivation and reduction of potential systemic toxicity. In A549 lung cancer cells, the albumin–Au catalyst mediated efficient intracellular phenanthridinium formation at concentrations as low as 0.65 μM, resulting in a marked reduction in cell viability. More recently, in 2023, the Jessen-Trefzer group reported a bimetallic protein-based nanoreactor containing ruthenium and gold, capable of catalyzing a two-step cascade synthesis of phenanthridine and phenanthridinium derivatives in aqueous media, highlighting the growing interest in multicatalytic systems for bioorthogonal chemistry.^38^

**Figure 7:**
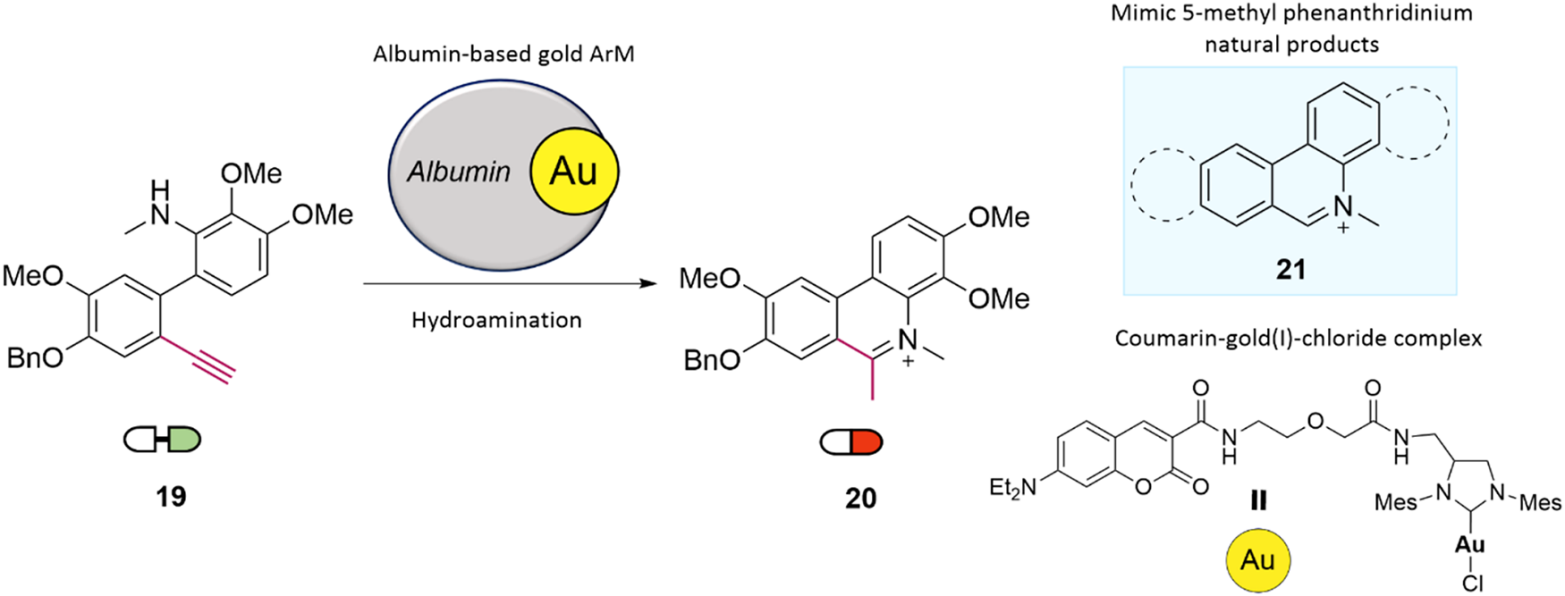
Synthesis of 4-methylphenanthridinium derivatives catalyzed by albumin–gold artificial metalloenzymes.

### 2.5 Quinoxalines

In 2021, Mascareñas and co-workers reported the in-cell synthesis of bioactive quinoxalines via a metal-carbene transfer reaction catalyzed by copper­(II) in live mammalian cells.^39^ This transformation involves the cyclization of ortho-aminoarylamines (22) with α-keto diazocarbenes (23), generating quinoxalines (24) through a sequence of N–H carbene insertion, intramolecular cyclization, and oxidative aromatization (Figure 8A). Among the products, 2-phenylbenzoquinoxaline (AG1385) was selected as a bioactive target due to its established inhibitory activity against tyrosine kinases and recognized antitumor potential (Figure 8B). The intracellular formation of AG1385 in HeLa cells induced greater cytotoxicity than the direct addition of the compound, which suffers from limited cellular uptake due to its hydrophobic nature. To enhance specificity, the authors further developed a copper-based catalyst conjugated to an integrin-targeting ligand, designed to selectively accumulate in integrin-overexpressing cell lines such as HeLa (Figure 8C). Cells were first incubated with the integrin-targeted copper catalyst, followed by washing and the sequential addition of the ortho-amino precursor (25) and diazo compound (26). This approach resulted in marked mitochondrial depolarization selectively in HeLa cells, which was attributed to the localized synthesis of AG1385 (27). In 2023, Palmans and colleagues extended this strategy by employing rhodium-catalyzed carbene transfer reactions for quinoxaline formation in live cells.^40^ To overcome solubility and stability challenges, they developed amphiphilic polymeric nanoparticles capable of encapsulating hydrophobic dirhodium carboxylate catalysts. This design enabled the in situ synthesis of fluorescent and cytotoxic benzoquinoxalines (e.g., AG1385) within HeLa cells at low catalyst loadings, demonstrating the potential of supramolecular encapsulation in bioorthogonal catalysis.

**Figure 8:**
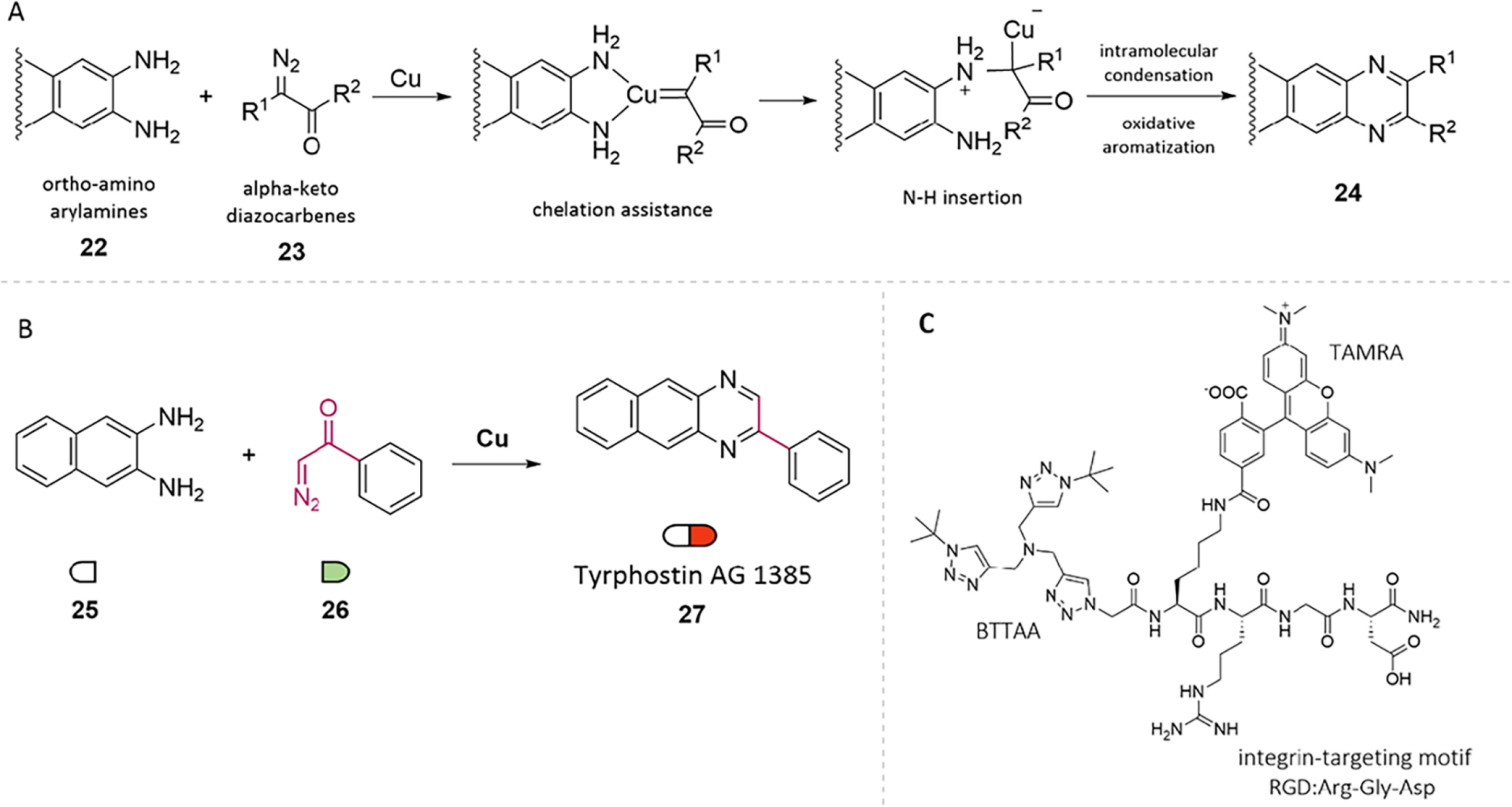
(A) In cellulo synthesis of quinoxalines by copper-promoted cyclization of *ortho*-aminoarylamines with α-keto diazocarbenes. (B) Bioorthogonal synthesis of Tyrphostin AG1385 (**27**) in cancer cells. (C) Structure of the integrin-targeted copper catalyst.

### 2.6 Coumarin

#### 2.6.1 Umbelliprenin

In 2019, Tanaka and co-workers developed a targeted bioorthogonal strategy for in-cell drug synthesis via ruthenium-catalyzed olefin metathesis by Grubbs-type artificial metalloenzymes (ArMs).^41^ The system was based on *N*-glycosylated albumin functionalized with α­(2,6)-sialic acid-terminated complex glycans, where a coumarin–Ru–Cl catalyst was anchored within the hydrophobic binding pocket (Figure 9A). This targeting strategy likely exploits the overexpression of galectin-8 in certain cancer cells, which exhibit a high affinity for α­(2,6)-sialic acid. As a model substrate, the natural coumarin umbelliprenin (29) was chosen as the drug target, a cytotoxic compound derived from Ferula species, which is recognized for its cytotoxic activity against various cancer cell lines.^42^ The corresponding prodrug (28), Pro-umbelliprenin, displayed strong binding affinity to the albumin carrier due to its hydrophobicity, which enhanced the catalytic performance. Cytotoxicity assays revealed that the combination of (28) and GArM-Ru significantly inhibited cell growth in SW620, HeLa, and A549 cell lines (Figure 9B).

**Figure 9:**
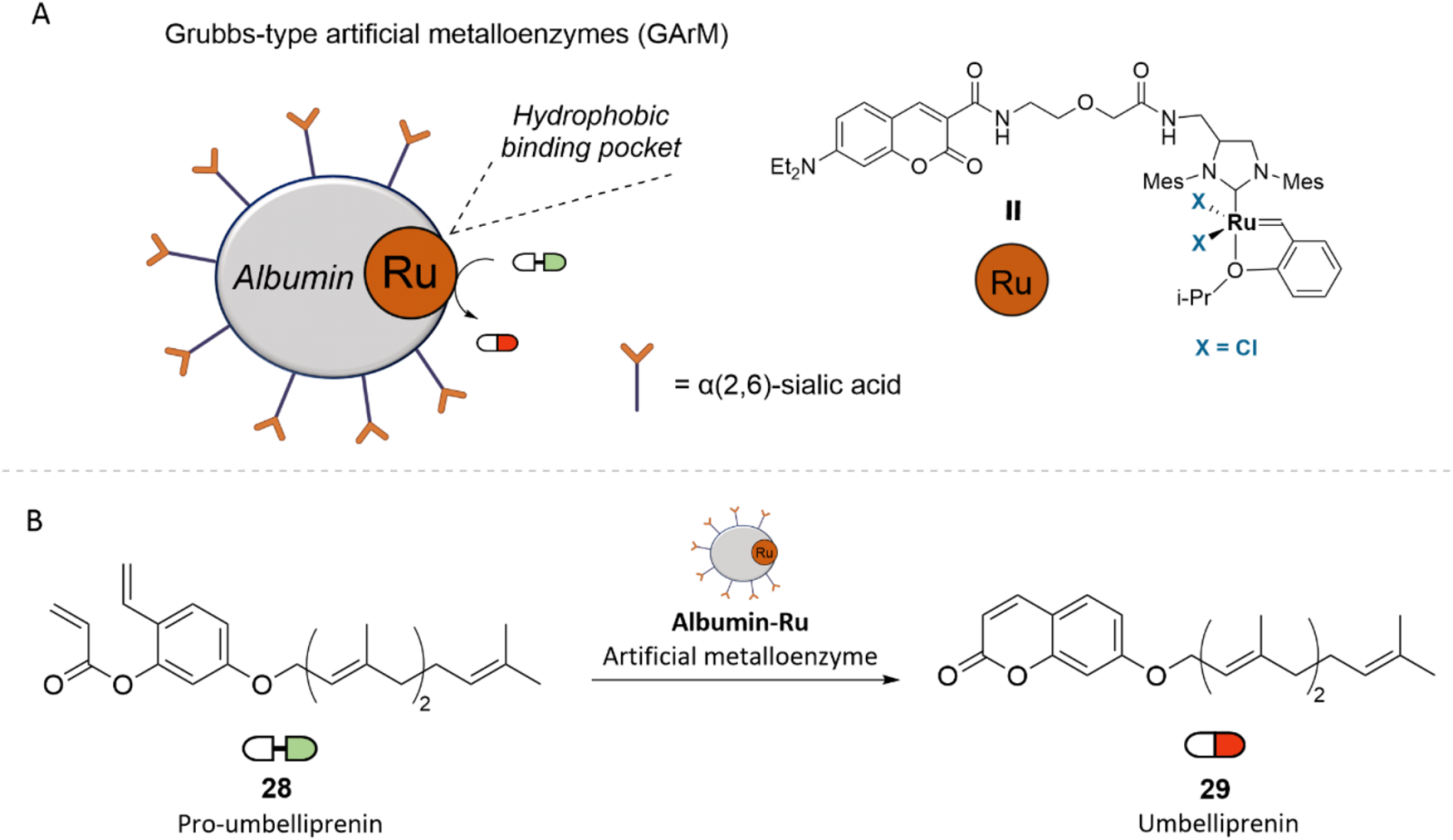
(A) Structure of a Grubbs-type artificial metalloenzyme. (B) In-cell synthesis of Umbelliprenin (**29**) via ruthenium-catalyzed olefin metathesis.

In 2025, Bai and co-workers further advanced ArM technology by engineering intracellular compartments in *E. coli* using HaloTag-SNAPTag fusion proteins to encapsulate Ru, Cu, and Pd catalysts. The ruthenium system enabled the bioorthogonal metathesis-based activation of Pro-umbelliprenin (28) in a colorectal cancer mouse model, showcasing the therapeutic applicability of bacterial ArMs in vivo.^43^

### 2.7 Naphthyl

#### 2.7.1 Combretastatin A4 Analog

In 2022, Tanaka and co-workers reported a prodrug strategy for the in-cell synthesis of phenyl-containing bioactive agents via ring-closing metathesis (RCM), catalyzed by their previously developed Grubbs-type ArMs (Figure 10A).^44^ The reaction is initiated with the formation of a cyclic intermediate (31), which undergoes aromatization to yield naphthyl derivatives (32). The leaving group significantly influenced the efficiency of this transformation, with acidic esters offering optimal reactivity. Based on these findings, the authors designed a pivalate prodrug (33) as a precursor to the potent naphthyl analog of Combretastatin A4 (CA4, 34), a well-known microtubule polymerization inhibitor (Figure 10B). Compound (34) exhibited nanomolar cytotoxicity in cancer cells, whereas its prodrug (33) was over 3000-fold less toxic in HeLa cells, highlighting its favorable safety profile. For targeted synthesis in cancer cells, GArM-Ru, an *N*-glycosylated albumin-based ArM, was employed, leveraging its affinity for galectin-8 in HeLa cells (Figure 9A).^41^ The therapeutic efficacy of (33) + GArM-Ru was validated in a murine tumor model.

**Figure 10:**
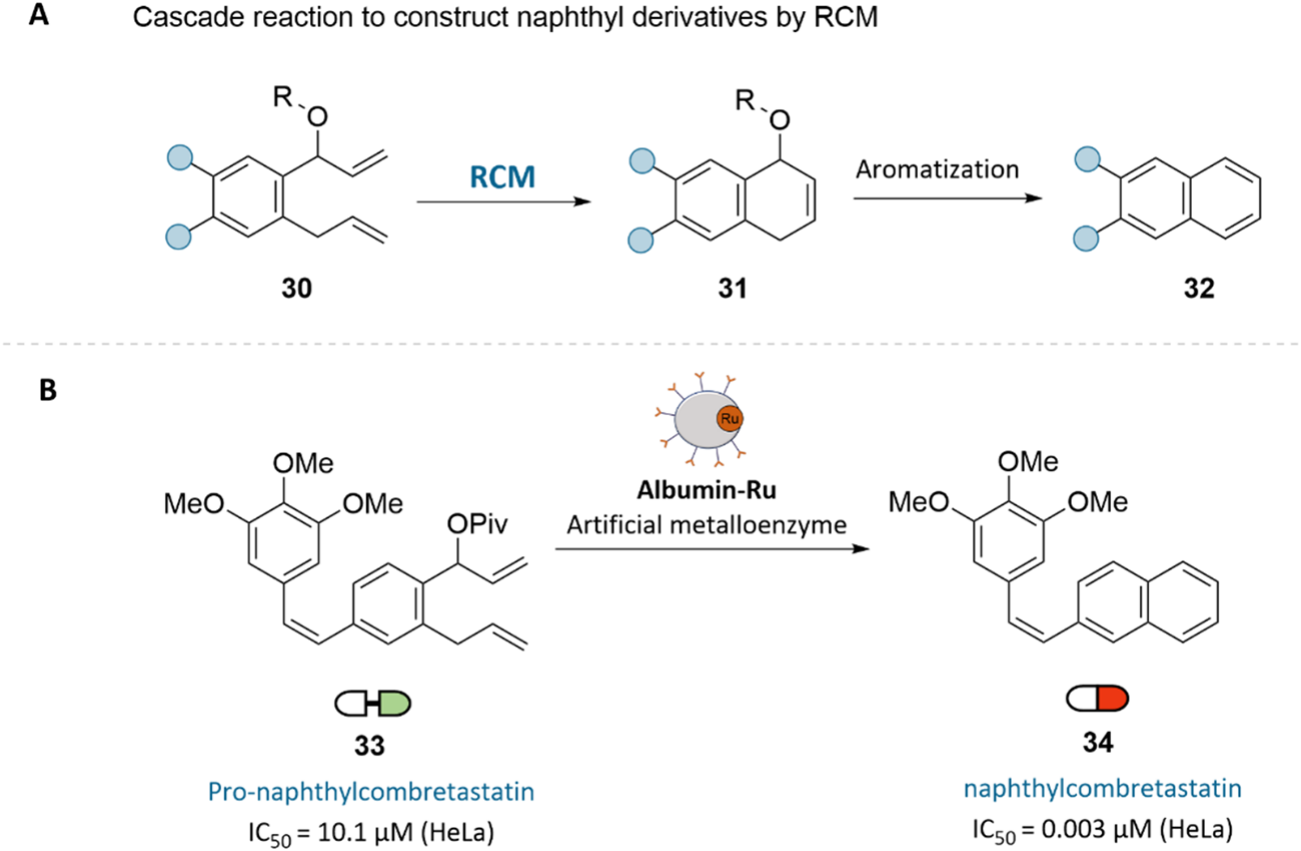
(A) Prodrug design for the cascade synthesis of naphthyl derivatives via ruthenium-catalyzed RCM. (B) Targeted bioorthogonal synthesis of Combretastatin A4 analog (**34**) using GArM-Ru.

In 2023, a second-generation platform was introduced based on a coumarin–Ru–I catalyst conjugated to the cRGD peptide and human serum albumin, aiming at integrin-overexpressing SW620 colon cancer cells.^45^ The iodide-ligated catalyst (Ru–I) was designed to enhance stability in blood, enabling efficient in vivo synthesis of the active drug (34) upon intravenous administration.

### 2.8 Pyrrolizidine Alkaloids (PAs)

Pyrrolizidine alkaloids (PAs) are a widespread class of natural products that are known for their toxicological relevance. Upon metabolic oxidation by cytochrome P450 enzymes, PAs are converted into dehydro-PAs (DHPs), which form electrophilic intermediates that covalently bind to cellular nucleophiles, inducing cytotoxicity and hepatotoxicity.^46^ In 2022, Yokoshima and co-workers reported the in situ bioorthogonal synthesis of DHP derivatives (36) from nontoxic homopropargylamine precursors (35), via gold-catalyzed 5-endo-dig cyclization (Figure 11A).^47^ In vitro assays in human cancer cell lines (HeLa, PC3, A549, and SW620) demonstrated that compound (35) was significantly less toxic than its cyclized counterpart (36), with up to a 25-fold difference in EC_50_. However, treatment with (35) in the presence of a coumarin–gold catalyst reproduced the cytotoxic profile of (36), confirming intracellular synthesis. Mechanistically, the strategy mimics the natural bioactivation of pyrrolizidines with gold-catalyzed cyclization generating reactive DHP species that form adducts with proteins and DNA (Figure 11B). Using a previously developed glycosylated albumin-based gold ArM (Alb-Au),^37^ the authors achieved selective activation in SW620 cells overexpressing galectin-8, while sparing A549 and HeLa cells. This selective cytotoxicity was attributed to the targeted delivery and localized synthesis of the DHP agent.

**Figure 11:**
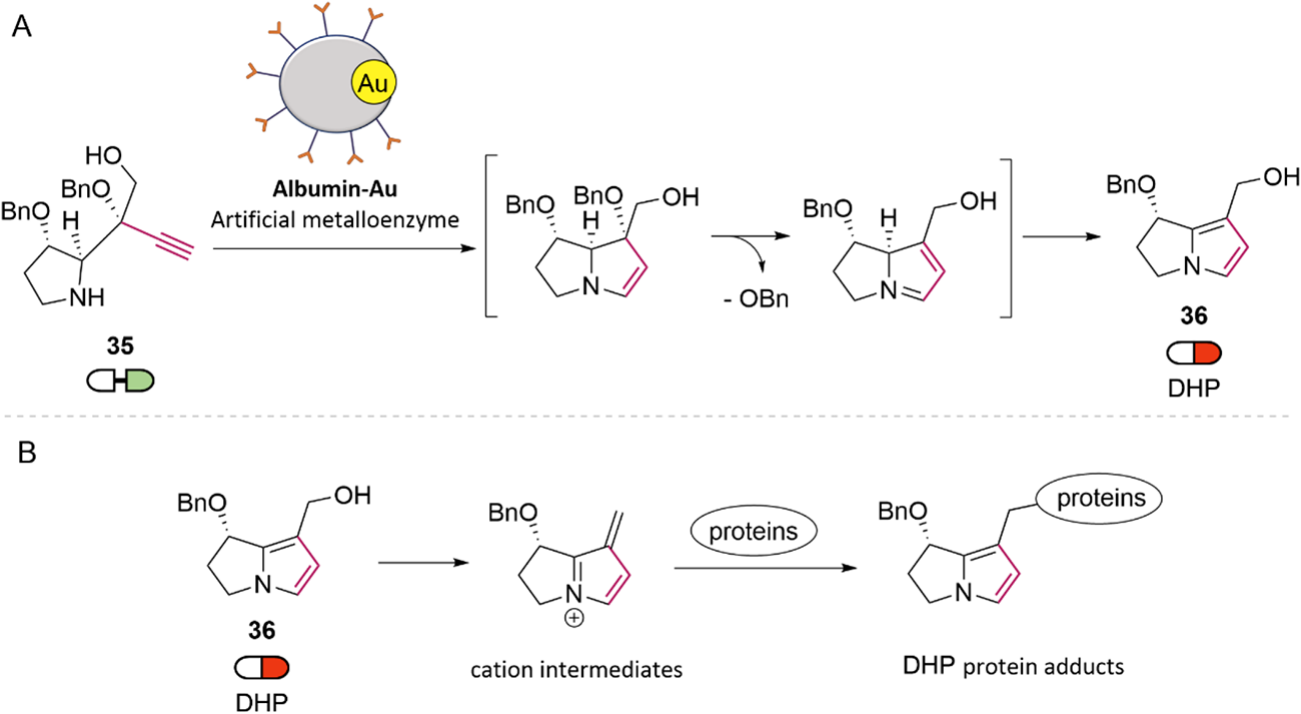
(A) In situ synthesis of dehydro-pyrrolizidine (**36**) catalyzed by albumin-based gold ArM. (B) DHP induces cytotoxicity via the electrophilic protein and DNA adduct formation.

## 3 Future Outlook

In summary, although a wide range of heterocycles display promising chemical diversity and biological activity, only a select few have been successfully synthesized in situ within cancer cells. This limitation stems from the need to adapt reactions to aqueous environments and to engineer novel bioorthogonal transformations and catalysts compatible with intracellular conditions. A promising strategy for expanding the repertoire of heterocycles in cancer therapy involves repurposing cyclization reactions originally developed for bioimaging, such as those forming indoles^48^ and thiazoles,^49^ for therapeutic applications. However, the design and synthesis of suitable precursors and reagents for these transformations remain challenging. These compounds often possess intricate molecular architectures that require a multistep synthesis and careful optimization to ensure bioorthogonality, stability, and selective activation. The overarching goal is to engineer nontoxic precursors that undergo efficient cyclization within the tumor microenvironment to generate cytotoxic heterocycles, thereby minimizing systemic toxicity associated with conventional chemotherapy. Ideally, such compounds should exhibit potent anticancer activity upon in situ activation with cytotoxicity in the nanomolar range. Addressing the current synthetic and biological challenges is essential to fully realizing the potential of in situ heterocycle formation in precision cancer therapy. Future advances in catalyst design, prodrug engineering, and targeting strategies are expected to significantly broaden the scope and impact of this emerging approach in medicinal chemistry.
